# Availability, utilisation and quality of maternal and neonatal health care services in Karamoja region, Uganda: a health facility-based survey

**DOI:** 10.1186/s12978-015-0018-7

**Published:** 2015-04-08

**Authors:** Calistus Wilunda, Koyejo Oyerinde, Giovanni Putoto, Peter Lochoro, Giovanni Dall’Oglio, Fabio Manenti, Giulia Segafredo, Andrea Atzori, Bart Criel, Alessio Panza, Gianluca Quaglio

**Affiliations:** Doctors with Africa CUAMM, Via San Francesco 126, 35121 Padua, Italy; Averting Maternal Death and Disability Program, Mailman School of Public Health, Columbia University, New York, NY 10032 USA; Doctors with Africa CAUMM, 7214, Kampala, Uganda; Institute of Tropical Medicine, Antwerp, Belgium; College of Public Health Sciences, Chulalongkorn University, Bangkok, Thailand; Department of Internal Medicine, Verona University Hospital, Verona, Italy

**Keywords:** Maternal health, Rural health, Emergency obstetric care, Uganda

## Abstract

**Background:**

Maternal mortality is persistently high in Uganda. Access to quality emergency obstetrics care (EmOC) is fundamental to reducing maternal and newborn deaths and is a possible way of achieving the target of the fifth millennium development goal. Karamoja region in north-eastern Uganda has consistently demonstrated the nation’s lowest scores on key development and health indicators and presents a substantial challenge to Uganda’s stability and poverty eradication ambitions. The objectives of this study were: to establish the availability of maternal and neonatal healthcare services at different levels of health units; to assess their utilisation; and to determine the quality of services provided.

**Methods:**

A cross sectional study of all health facilities in Napak and Moroto districts was conducted in 2010. Data were collected by reviewing clinical records and registers, interviewing staff and women attending antenatal and postnatal clinics, and by observation. Data were summarized using frequencies and percentages and EmOC indicators were calculated.

**Results:**

There were gaps in the availability of essential infrastructure, equipment, supplies, drugs and staff for maternal and neonatal care particularly at health centres (HCs). Utilisation of the available antenatal, intrapartum, and postnatal care services was low. In addition, there were gaps in the quality of care received across these services. Two hospitals, each located in the study districts, qualified as comprehensive EmOC facilities. The number of EmOC facilities per 500,000 population was 3.7. None of the HCs met the criteria for basic EmOC. Assisted vaginal delivery and removal of retained products were the most frequently missing signal functions. Direct obstetric case fatality rate was 3%, the met need for EmOC was 9.9%, and 1.7% of expected deliveries were carried out by caesarean section.

**Conclusions:**

To reduce maternal and newborn morbidity and mortality in Karamoja region, there is a need to increase the availability and the accessibility of skilled birth care, address the low utilisation of maternity services and improve the quality of care rendered. There is also a need to improve the availability and accessibility of EmOC services, with particular attention to basic EmOC.

## Introduction

Uganda has an estimated population of 35.8 million people and is experiencing a rapid population growth. It is a developing country with an estimated per capita income of US$ 510 and a life expectancy at birth of 54 years [1]. The HIV prevalence among the adult population (15–49 years) is about 6.4% [2]. Indicators of maternal and neonatal health (MNH) in the country are also poor. The Maternal Mortality Ratio (MMR) per 100,000 live births is persistently high despite falling from 571 in 1990, to 505 in 2000, and to 438 in 2011 [3,4]. The infant mortality rate is estimated to be 54 per 1,000 live births [3]. Uganda signed on to the Millennium Development Goals (MDGs) of which the targets of the fifth MDG (MDG 5) are to reduce the MMR by 75%, between 1990 and 2015, and to increase coverage of skilled attendance at birth to 95% by 2015 [5]. Achieving these targets in Uganda is challenging because of many barriers to accessing health care as reflected in institutional delivery rate which remains unacceptably low in spite of increases in recent years: 37% in 2001, 42% in 2006 and 57% in 2011 [3].

Barriers to accessing health care services in Uganda include: i) financial limitations; ii) poor geographic accessibility of health facilities in terms of transport and distance; iii) lack of decision making power among women; iv) inability to afford the medical supplies that are often compulsory at public health facilities; v) bad attitude of health workers – including neglect and abuse, and vi) preference for traditional birth attendants (TBAs) [6,7].

Access to quality emergency obstetrics care (EmOC) is fundamental to reducing maternal and newborn mortality rates [8] and is thought to be a possible way of achieving the MDG 5 target [9]. Yet, a national survey conducted to determine availability of EmOC in Uganda concluded that, among health facilities expected to offer basic EmOC, 97.2% were not offering the service. In addition, severe shortcomings in the quality of care were noted [10]. Recent data suggests that there is poor utilisation antenatal care (ANC) in Uganda as only 47% of pregnant women attend four ANC visits [11]. Perhaps the poor ANC coverage contributes in part to the 18% of births assisted by TBAs, 15% by relatives or friends, and 7% without any assistance [3].

The Karamoja region in north-eastern Uganda occupies an area of 35,007 km^2^ and has a population of 1,294,000 according to 2012 projections [12]. The region has consistently demonstrated the nation’s lowest scores on key development and health indicators and presents a substantive challenge to Uganda’s stability and poverty eradication ambitions. Over 70% of the population experiences critical food insecurity. In addition to this, decades of armed conflict due to internal and cross-border cattle raiding has resulted in high levels of civil unrest. In attempts to reduce conflict in the region, the government has instituted a policy framework that promotes disarmament and encourages sedentary life styles in place of mobility which is traditionally a characteristic of the region’s inhabitants [13]. A combination of different factors severely compromises the effectiveness of MNH care in Karamoja. These include the break-down of the formal health care system, the increased frequency of epidemics, the loss of adult family members to violent death, starvation, outward migration, the disruption of formal marriage structures and the increasing problem of alcohol abuse [14]. In this region for example, coverage for skilled attendance at birth is only 31% compared to the national average of 58% [3].

Doctors with Africa-CUAMM, has been operating in Karamoja for about 30 years; working with district health offices and health facilities to strengthen health systems and to improve access to quality health care [15]. In order to improve planning, monitoring and evaluation of health interventions, Moroto District Health Office in collaboration with Doctors with Africa-CUAMM conducted an in-depth assessment of all health facilities providing MNH services in Moroto district (currently Moroto and Napak districts). The objectives of this study were: i) to establish the availability of MNH care services at different levels of health units; ii) to assess their utilisation; and iii) to determine the quality of services provided.

## Methods

### Study setting

The study was conducted in March 2010 in the Moroto District of Karamoja, which at the time had a population of 276,000 [16]. Shortly after the study period, the district was split into two districts: Moroto and Napak. Karamoja region currently has five other districts: Abim, Amudat, Kaabong, Kotido and Nakapiripirit. The Ugandan health care infrastructure includes village health teams (the lowest level or level I), HCs and hospitals (district/rural, regional and national hospitals). HCs are graded as II, III, or IV, according to the administrative zone served and by the types of services that they provide [17]. HC II provides outpatient care, ANC, immunization and outreach programmes. HC III provides all the services of HC II, plus inpatient care and environmental health. A HC III should be able to function as a basic EmOC (BEmOC) facility [18]. HC IV provides all the services of HCs III, plus surgery, supervises the HCs II and III, and in theory, should be able to function as a comprehensive EmOC (CEmOC) facility [18].

A BEmOC facility is one that is performing all of the following 7 signal functions: i) administration of injectable antibiotics; ii) administration of oxytocic drugs; iii) administration of anticonvulsants; iv) manual removal of the placenta; v) removal of retained products (e.g. manual vacuum aspiration); vi) assisted vaginal deliveries (e.g. vacuum extraction); and vii) neonatal resuscitation with bag and mask [19]. A comprehensive EmOC facility is one that is performing all signal functions in BEmOC as well as caesarean sections and blood transfusions [19]. In Uganda, MNH services offered at public health facilities are officially free of charge; however, due to frequent shortages of drugs and supplies, patients are sometimes requested to procure missing items.

### Study design

A cross sectional study design at health facility level was used. Data collection tools were adopted mainly from the Safe Motherhood Needs Assessment manual [20] and were locally adapted and pretested. They can be divided into three different pillars: i) Facilities/Health personnel (facility inventory checklist and maternity nurses/midwives interview); ii) Clinical (normal delivery record review, caesarean section record review, eclampsia record review and obstructed labour record review) and iii) Outpatient (antenatal client exit interviews, antenatal client record review, post natal exit interview). Table 1 describes the utilized tools, the target and achieved sample size for each tool and the focus of the tool.

**Table 1:** **A description of tools used and the purpose of each tool**

	**Data collection tool**	**Sample size**		**Focus of data collection**
		**Target**	**Achieved**	
**Facility**/ **Staffing**	Facility management	20	20	Management of services; staffing; services provided; EmOC services; availability of registers/cards (neonatal register, delivery register, ANC register, family planning register, etc.); key infrastructure; equipment, consumables, essential drugs; facility statistics etc.
	Midwife or maternity nurse interview	-	29	Practice of midwifery skills by midwives/nurses; knowledge on maternal and neonatal care; recent nurse/midwife practice of life-saving skills
**Clinical**	Normal delivery record review	334	337	Assessment of quality of normal delivery practice using indicators of performance.
	Caesarean section record	140	140	Speed of caesarean section efficiency of caesarean section service; quality of post caesarean section care
	Eclampsia record review	--	9	Indicators of good management of eclampsia
	Obstructed labour record review	57	57	Outcomes of obstructed labour
**Outpatient**	Antenatal care exit interview	384	347	Services received by antenatal women on the day of the visit or at any time during the current pregnancy; services received during any antenatal visits; knowledge of warning signs during pregnancy.
	Antenatal client record review	384	332	Services received by antenatal clients: Intermittent preventive therapy iron/folic supplementation, tetanus toxoid administration, insecticide-treated bed net provision, provision of de-worming medication, syphilis test, haemoglobin test, etc.
	Postnatal exit interview	379	215	Length of stay at the health facility after delivery; timing after delivery of postpartum visit; postpartum services provided

### Sample size, sampling and data analysis

Sample sizes for antenatal client exit interviews and record reviews, normal delivery record reviews, obstructed labour record reviews, postnatal care interviews and caesarean section record reviews were determined using the Taro Yamane formula for a finite population based on data from the routine health information system [21]. The calculated samples were allocated to health facilities in proportion to the number of clients served. For a review of normal deliveries, obstructed labour cases, and caesarean sections, records of deliveries that took place in the past year were systematically sampled. Systematic sampling was also done for antenatal and postnatal exit interviews and antenatal record reviews, although in some cases, the number of clients was insufficient to allow for any sampling and therefore all available clients were interviewed. On average, 5 maternity nurses/midwives per health facility were interviewed. All available records of eclampsia deliveries in the past one year were reviewed. The expected number of deliveries per year (used as denominator for caesarean section rate, ANC coverage, intermittent preventive therapy (IPT) for malaria and postnatal care (PNC) coverage) was estimated by multiplying the crude birth rate (CBR) by the population of the two districts. The CBR was taken to be 4.85% according to Uganda’s Health Management Information System manual [22]. Service statistics for facilities over the previous year (2009) were extracted from maternity registers. The items counted were vaginal and caesarean deliveries, direct and indirect obstetric complications and direct and indirect maternal deaths. These data were used to calculate EmOC indicators by following standard United Nations (UN) guidelines [19].

The survey sought to collect information on the number of key health workers related to maternal and neonatal health and to assess their retention by estimating the duration of service at their duty stations. This was done for selected staff cadres and was obtained by dividing the number of person months at each duty station by the number of staff whose data on duration of service at duty station was available. Data were collected by two survey teams, each consisting of four trained data collectors and a supervisor. Data entry and analysis were carried out using SPSS version 16. Analysis was done mainly by way of descriptive statistics. The study was approved by the Ugandan National Council for Science and Technology and the Moroto District Health Management Team. Interviewees provided informed consent either by signing or thumb-printing on the consent form.

## Results

### Availability of maternal and neonatal health care services

Moroto and Napak districts combined had 2 general hospitals, 10 HC IIIs and 8 HC IIs. There were no HC IVs. One HC II was in the process of being upgraded to a HC III (and hence was already conducting deliveries).

Normal delivery services were available at only 65% of health facilities; 2 hospitals, 10 HC IIIs and 1 HC II. The two hospitals functioned as full CEmOC facilities; they performed all 9 signal functions in the 3-month period preceding the survey (Table 2). The number of CEmOC facilities per 500,000 population was 3.7 (2/276,000). Each district had a CEmOC facility.

**Table 2:** **Number of facilities by emergency obstetric care signal functions**

**Characteristic**	**Type of facility**		**Total**
	**Hospital**	Health centre*	
Number of health units	2	11	13
Services provided within the past 3 months (EmOC signal functions)			
1. Parental antibiotics	2	11	13
2. Parental oxytocics	2	10	12
3. Parental sedatives/anticonvulsants	2	10	12
4. Manual removal of placenta	2	7	9
5. Removal of retained products	2	4	6
6. Assisted vaginal delivery	2	0	2
7. Neonatal resuscitation with bag and mask	2	5	7
8. Blood transfusion provided	2	NA	2
9. Caesarean section	2	NA	2
Current EmOC Status			
Comprehensive EmOC^a^	2	NA	2
Basic-EmOC^b^	0	0	0
Non EmOC^c^	0	11	11

*Health centres providing delivery service (10 HC III and 1 HC II).

^a^if all 1–9 were provided, ^b^if only 1–7 were provided, ^c^if any of 1–7 was not provided. NA: Not applicable.

Among HCs that conducted deliveries, none provided all the 7 BEmOC signal functions 3 months prior to the survey (Table 2). The signal functions which require specific manual skills and specific equipment were the least available. No HCs performed vacuum extractions, 18% performed manual vacuum aspiration, and 64% performed manual removal of the placenta within the 3-month period. Almost all the HCs administered injectable anticonvulsants, oxytocics and antibiotics. Five (45%) of the HCs missed 1 or 2 BEmOC signal functions while the rest (55%) missed more than 2 signal functions. Reasons for not having performed signal functions were sought and multiple responses were given (Table 3). Lack of supplies/equipment was the most frequently mentioned reasons for non-performance of signal functions. ANC services, postnatal care and family planning services were available at 75%, 65% and 50% of the health facilities, respectively (Table 4).

**Table 3:** **Reasons for not performing signal functions at health centres**

**Signal function**	**No. of HCs that did not perform signal function**	**Number of HCs that did not perform signal function due to** ^**a**^ **:**		
		**Lack of trained staff**	**Supplies/ equipment**	**No indication**
Parenteral oxytocics	1	0	0	1
Manual removal of placenta	4	2	0	2
Neonatal resuscitation	6	0	6	2
Removal of retained products	7	6	7	1
Assisted vaginal delivery	11	5	9	2
Parenteral anticonvulsants	1	0	1	0
HCs mentioning the reason for non-performance of any of the functions		13	23	8

^a^ Multiple responses allowed.

Reasons for not performing signal functions were classified as follows.

a. Lack of trained staff 1) Required health workers are not posted to this facility in adequate numbers (or at all) 2) Authorized cadre is available, but not trained. 3) Providers lack confidence in their own skills. b. Supplies/Equipment Issue. 1). Supplies/equipment are not available, not functional, or broken. 2) Needed drugs are unavailable. c. No Indication - no client needing this procedure came to the facility during this time period.

**Table 4:** **Availability and utilisation of maternal and neonatal health over a 3 month period**

**Indicator**	**Value**	**Minimum acceptable**
Population	276000	-
Expected number of deliveries in 2009	13386	-
CEmOC facilities per 500,000 population	3.7 (2/276000)	1
BEmOC facilities per 500,000 population	0.0 (0/276000)	4
Met need for EmOC services	9.9% (198/2008)	100%
Direct obstetric case fatality rate	3% (6/198)	<1%
Deliveries conducted in EmOC facilities	10.1% (1352/13386)	15%
Deliveries conducted in all health facilities	15.4% (2055/13386)	-
Population based caesarean section rate	1.7% (229/13386)	5%
Antenatal care availability	75% (15/20)	-
Antenatal care 1 visit	61.9% (8288/13386)	-
Antenatal care 4 visits	31.7% (4238/13386)	-
Intermittent preventive therapy 1st dose	47.7% (6386/13386)	-
Intermittent preventive therapy 2nd dose	27.7% (3714/13386)	-
Postnatal care availability	65% (13/20)	-
Postnatal care coverage	31.3% (4187/13386)	-
Health facilities with family planning services	50% (10/20)	-

### Utilisation of maternal and neonatal health care services

The institutional delivery rate in the study districts combined was 15.4% (Table 4). The population-based caesarean section rate was 1.7% and the met need for EmOC (total complications managed in an EmOC facility as a proportion of expected complicated deliveries in a given population) in the study area was 9.9%. Only 31.7% of women attended at least 4 ANC visits and only 27.7% received two doses of intermittent preventive therapy (IPT) for malaria.

### Quality of maternal and neonatal health care services

The case fatality rate was calculated for those facilities that qualified as CEmOC units and registered maternal deaths. The case fatality rate for direct obstetric complications was 3% compared to a maximum of 1% currently acceptable by the UN (Table 4).

### Facility inventory checklist

Among facilities offering delivery services (n = 13), 54% had a waiting area for maternity clients. A similar percentage of health facilities (54%) had a private examination room, 38% had running water near consultation rooms, 77% had a refrigerator, 23% had an incinerator, and 62% had a toilet. Additionally, 15% of the facilities had a maternity waiting home, 69% had a bed for gynaecological examinations and 54% had a delivery/labour room with a bed. Radio communication systems or a mobile phone, and a car ambulance were available at 46% of the facilities. In general, hospitals had better infrastructure than HCs. Regarding maternity equipment, hospitals were well equipped but deficiencies were noted in HCs whereby 50% lacked basic equipment for normal delivery (scissors, suture materials and a long needle holder). Some HCs lacked equipment for neonatal resuscitation. Hospitals were well equipped with consumable supplies and drugs while health centres had deficiencies. All facilities conducting deliveries had delivery registers. Neonatal registers were available in 85% of the health units. Among facilities offering ANC, 93% had ANC registers while among those providing family planning, only one did not have a family planning register.

### Staffing

Assessment of staffing (according to staffing norms for MNH) was limited to midwives. In total, the district had a shortage of 47 midwives representing half of the minimum required number. The average duration of service of staff at their present duty stations was assessed. The mean duration was 51 months as compared to 62, 59, and 12 months for clinical officers, midwives/nurses and doctors, respectively.

### Maternity staff interview

Practice of midwifery skills in the previous three months was assessed among 29 nurses and midwives working in the maternity wards. All the staff had practiced intravenous infusions while 90% had practiced focused ANC. About 70% had performed cervical examinations, sutured an episiotomy, used a partograph to monitor labour, performed neonatal resuscitation and sutured vaginal lacerations. Manual removal of the placenta had also been done by 62% of the staff. Skills which were practiced by the least proportion of staff were, performing pap smears and sutures of 3rd/4th degree lacerations, suture of cervical lacerations, vacuum extractions and manual vacuum aspiration (each about 25%). Staff members in hospitals were more likely to have practiced these skills compared to their counterparts at HCs.

The knowledge of maternity staff on various aspects of MNH with a special focus on emergencies was also assessed. Each question had a set of essential solutions that were considered to be sufficient. Seventy nine percent of the staff mentioned all essential aspects of prevention of maternal to child transmission (PMTCT) of HIV. All essential actions in management of postpartum haemorrhage (PPH) and actions to take if a newborn failed to breathe were mentioned by 62% of the interviewees. Actions to be taken for the monitoring of labour and actions to be taken for immediate care of new born were mentioned correctly by 55% and 52% of staff respectively. Less than 50% of the staff mentioned essential actions in management of newborn sepsis; low birth weight babies; incomplete abortion; retained placenta; women with general malaise 24 hours after delivery, and signs of postpartum haemorrhage. In general, staff members in hospitals tended to perform better than those in HCs.

Practice of life saving skills in the past three months was also assessed. About 66% of maternity staff had managed obstructed labour, 45% had managed post-partum haemorrhage, 48% had managed abortion complications, 52% had managed puerperal sepsis and 35% had managed eclampsia. Staff members working in hospitals were more likely to have managed obstetric complications compared to their counterparts in HCs. Postpartum haemorrhage had been managed by only 15% of staff in HCs compared to 89% of staff at hospitals.

### Antenatal client exit interview

Three hundred and forty seven women attending ANC were interviewed (mean age 26 years). Almost all the women (99%) had come to their respective facilities by foot (median walking time 60 minutes, inter-quartile range 30–90). A quarter of the women had a gravidity of >4 and 28.5% were primigravidae. Only 1% did not have any ANC record (either a card or a book). The study investigated services received by antenatal women on the day of the visit or at any time during the current pregnancy. Concerning services received on the day of the visit, the Mother-Baby Package identifies key services that should be provided at every antenatal visit: abdominal examination, foetal heartbeat monitoring and blood pressure recording. These three services were provided to 94%, 89% and 61% of clients, respectively. Only 57% of women had received all the 3 key services. Additionally, antenatal clients were asked if they had received any of the specified services at any time during the current pregnancy. The results are presented in Table 5. Concerning the knowledge of warning signs during pregnancy among antenatal clients, severe vaginal bleeding was mentioned most frequently (57%) followed by postpartum sepsis and premature rupture of membranes (42% each). Prolonged labour was reported by 8% of the respondents. A majority of the respondents (62%) mentioned at least 3 danger signs.

**Table 5:** **Frequency and percent of women by antenatal activities carried out at any visit during the current pregnancy**

**Activity**	**Number (n = 347)**	**% (95% CI)**
Received iron supplements	318	92 (88–94)
Blood sample taken	302	87 (83–90)
Sexually transmitted diseases, HIV/AIDS talked about	278	80 (76–84)
Advice on how to take care of your baby provided	265	76 (72–81)
Benefit of birth in the health facility discussed	263	76 (71–80)
Medical history taken	252	73 (68–77)
Family planning discussed	239	69 (64–74)
What to do if there is a problem with pregnancy discussed	238	69 (64–73)
Place of birth discussed	236	68 (63–73)
Information about diet and nutrition provided	227	65 (60–70)
Urine sample taken	25	7 (5–11)

### Antenatal client record review

Three hundred and thirty two records of women attending the ANC clinics were reviewed. The following services had been recorded: provision of IPT (93%); HIV tests (89%); iron/folic supplementation (88%) and tetanus toxoid administration (81%). Other services recorded were insecticide-treated bed net provision (55%), provision of de-worming medication (51%), syphilis tests (4%) and results of haemoglobin tests (1%). The results of syphilis tests were not available on records of all clients attending care at HCs.

### Postnatal care exit interview

Data were collected from 215 women attending postnatal clinics. Their median parity was three. Less than a quarter of the women (23%) had babies aged one week old but a majority (70%) had babies aged more than four weeks. Most of them (88%) attended their first postnatal visit in less than 1 week after delivery but 7% did so more than four weeks after delivery. This means that most of the women had attended the postnatal clinic more than once after delivery. Less than a third (30%) of the women gave birth to the current baby at a health facility. Of all, 87% reported that their baby was examined, 58% discussed with the health worker on how to care for the baby and 58% said breastfeeding was discussed. Only 62% of the women had been given vitamin A tablets and 50% had been talked to about family planning. Only 2% of the women had asked any question to the provider and 6% had felt they had been given a chance to be involved in care provided. A majority (91%) had been asked to come back. Information about having received a blood pressure check, an abdominal examination, a vaginal examination and inquiry about abnormal bleeding during postpartum visits were collected from women with children aged less than one month (65 subjects). Among these, 15% had their blood pressure checked, 25% had an abdominal examination, 17% had a vaginal examination and 22% were asked about abnormal bleeding.

### Normal delivery record review

Records of 273 women were reviewed. More than a half (54%) delivered within one hour of admission; 24% in two to four hours; 14% in five to six hours; and 8% after seven hours. Regarding the length of stay at the health facility after delivery, about half of them (53%) left the facility one day after delivery; 45% left on the day of delivery and only 2% left after more than one day. Table 6 shows the frequency and percent of normal delivery records with indicated delivery practice recorded. The most frequently recorded items were oxytocic administration (90%) and status of the placenta and membranes (89%) while the least frequently recorded items were condition of lochia (28%) and at least hourly blood pressure measurement (16%).

**Table 6:** **Frequency and percent of normal delivery records with indicated delivery practice recorded**

**Action recorded**	**Number (n = 273)**	**% (95% CI)**
Mother received oxytocic after delivery	245	90 (86–93)
Status of the placenta and membranes	244	89 (85–93)
Birth weight*	289	86 (82–89)
Amount of blood loss	229	84 (79–88)
Any APGAR score*	275	82 (77–85)
Vaginal examination at least 1 every 4 hours	187	69 (63–74)
Foetal heart rate at least hourly	161	59 (53–65)
Pelvic exam done on admission	111	41 (35–47)
Post-delivery blood pressure of mother	107	39 (34–45)
Temperature checked on admission	106	39 (33–45)
Post-delivery pulse of mother	106	39 (33–45)
Post-delivery mother’s temperature	105	39 (33–44)
Uterine involution	98	36 (30–42)
Condition of lochia	77	28 (23–34)
Blood pressure at least hourly	45	16 (13–21)

*n = 337.

### Caesarean section record review

A total of 140 emergency caesarean section records were reviewed. Cervical dilatation had been recorded in 68% of the records. Cephalopelvic disproportion/prolonged labour was the most common indication for caesarean (51%) followed by previous scaring (14%). Other indications were placenta previa/abruption (8%), cord prolapse (6%), foetal distress (5%), breech/malpresentation (5%) and others (11%). Labour monitoring using a partograph had been done in only 29% of these deliveries. The “decision to incision” interval was less than 60 minutes for 76% of women; one hour for 15% and more than one hour for 9% of the cases. Administration of antibiotics had been recorded on most of the charts (89%) but it could not be ascertained, for every record, whether this was done before or after caesarean, or before or after signs of infection. In 9% of the cases reviewed, there was wound infection. The mean duration of hospital stay after caesarean was eight days with most of the women (62%) staying for 7–8 days. There was only one caesarean record (1%) with a maternal death. Foetal outcomes were: live birth (89%), fresh stillbirth (10%) and macerated stillbirth (1%). Foetal outcomes were also assessed according to indication of caesarean. High risks of stillbirth were in placenta previa/abruption (5/11, 46%) and cord prolapse/malpresentation (3/8, 38%).

### Eclampsia record review

Only nine records of eclampsia were encountered. In more than half of the records (56%), no drugs were administered. Antihypertensives were administered in a third of the records while an anticonvulsant (diazepam or magnesium sulphate) was administered in 44%. In 67%, neither blood pressure nor foetal heart beat were checked hourly. In none of the records were both blood pressure and foetal heart beat checked hourly.

### Obstructed labour record review

Fifty seven obstructed labour cases were reviewed. Most (77%) were delivered by caesarean section and 21% by vacuum extraction. Regarding foetal outcomes, 75% of cases resulted in a live birth in good condition (APGAR score 7–10 at 1 minute) while the rest were live births but with a APGAR score <7. Caesarean section was performed within 1 hour of the action line in 52% of the records.

## Discussion

### Availability

The current study shows important aspects of maternal and newborn services provided in the study area. Moroto and Napak districts, combined, exceed the minimum requirement for availability of CEmOC facilities which is 1 per 500,000 population [23]. This is justified given that the districts are very sparsely populated with the services being provided only in the two available hospitals. The population-based caesarean section rate, which is an indicator of accessibility and utilisation of EmOC, is low despite an overall adequate number of CEmOC facilities. This can partly be explained by the sparseness of the population in the districts, poor infrastructure and lack of reliable transportation resulting in poor geographical accessibility to these facilities [24]. The prevalence of high poverty levels in the area does not permit many women to pay for available ambulance services/other means of transportation or to buy supplies/drugs, which are sometimes missing at the facilities [24]. The minimum requirement of five EmOC facilities with at least one CEmOC facility per 500,000 population was not met in the districts. Without an efficient referral system to the hospitals, the lives of mothers and newborns delivering at the HCs is at great risk. Many maternal deaths can be averted if skills to perform assisted vaginal deliveries and removal of retained products are available to women in HCs. The adequate number of CEmOC facilities and inadequate number of BEmOC facilities reported in this study seems to be a common finding in many EmOC surveys [10,25-29]. It is of vital importance to upgrade some HC IIIs to provide BEmOC. However, the utilisation of the available EmOC facilities is still sub-optimal which calls for demand creation. Given that an estimated 74% of maternal deaths could be averted if women have access to interventions for preventing and treating pregnancy and birth complications [8,9], improving the availability of EmOC services is critical to reducing maternal and neonatal mortality.

### Utilisation

Geographical access to health services in Uganda has reportedly improved from 49% to 72% of the population living within 5 km of a health facility, in the period 1990–2005 [30,31]. This would be expected to yield some improvement in access [32]. However, the improvement in geographic accessibility at national level has not resulted in significant levels of utilisation in the study area. The institutional delivery rate of 15% in the study districts is low when compared with the 2011 Ugandan Demographic and Health Survey (DHS) finding of 27% for the Karamoja region [3]. This could be due to the dynamic nature of the population given that it is composed primarily of nomadic pastoralists. The percentage of deliveries conducted in EmOC facilities is lower than the recommended minimum of 15% [23]. In addition, the met need is less than the recommended 100%; all of these factors are indications of poor utilisation of health facilities for maternity services in the study area. A recent review has found that the met need for EmOC is negatively correlated with maternal mortality [33]; further highlighting the critical role of EmOC in maternal mortality reduction.

More efforts are needed to increase uptake of delivery and postnatal care, because most maternal deaths occur during and after delivery [34]. Other studies have shown that lack of knowledge/awareness, perceived poor quality of services, lack of confidentiality and poor attitude of health workers are barriers to health service utilisation [32]. On the other hand, interventions such as the removal of user-fees in government facilities have led to increases in the utilisation of services [35] and the government continues to make an effort to improve the availability of health services [36]. A qualitative study in Moroto and Napak districts found that the main barriers to utilisation of institutional delivery care were outside the scope of the health sector, and called for a multi-sectorial approach in tackling the problem [24]. In order to improve access, the government and development partners will need to continue removing barriers on both the demand and supply sides and address indirect barriers that are outside the scope of the health sector such as insecurity, poverty and shortage of food in the study area [24].

### Quality of care

It is recommended that pregnant women attend a minimum of four ANC visits to allow for appropriate delivery of a complete package of ANC [37]. Utilisation of ANC services is very low in Moroto and Napak districts when compared with the national averages: 62% versus 94% for at least one ANC visit and 32% versus 47% for at least 4 ANC visits [3]. A significant proportion of antenatal clients did not receive all the 3 Mother-Baby Package services, signaling quality gaps in service delivery. Such missed opportunities have been found in other studies in Uganda [38,39].

The prevalence of knowledge of at least three key danger signs during pregnancy among antenatal clients was 62%. Compared with other surveys [39,40], this is not bad. However, prolonged labour (which is one of major causes of maternal mortality in low-income countries) was only reported by 8% of respondents, as reported in other studies [39,40]. Knowledge of key danger signs is essential in prompting women to seek skilled attendance at birth and also to seek referral in case of complications [39,41].

About 31% of mothers who delivered in the study districts visited health facilities for postnatal care. Most mothers who attend these postnatal visits do so during the first week of delivery, probably at the same time when they bring their infants for immunization. At the time of this study, attendance of postnatal visits was being boosted by the associated food distribution; casting doubts on sustainability.

A review of delivery records revealed gaps in quality of intrapartum care. A partograph provides objective data to monitor maternal and foetal wellbeing during labour and serves as a basis for timely clinical decisions to save the life of the mother and/or the foetus [42]. Yet in this study, a number of clients were not monitored using partographs. Even among those with completed partographs, some were not monitored according to the norm. This may reflect inappropriate management of labour and/or poor use of the instrument. Lack of blank partographs in some health facilities, lack of training in the use of partographs and the fact that some women arrive at the health units while in the 2^nd^ stage of labour could explain why labour monitoring using partographs was not done [43]. A review of immediate postnatal care records revealed gaps in the technical quality of postnatal care provided. Very few women attending postnatal visits had asked any question to the provider or felt they had been given a chance to be involved in care provided. This is a reflection of lack of patient-centred care and poor quality of provider-patient interaction.

A review of management of obstructed labour cases showed that among cases that ended up in caesarean sections, it was only in half of the cases that the intervention was conducted within one hour past the action line. This is an aspect of the third delay (delay in receiving service within health facilities) of the three delays model [43]. This delay should be viewed as a problem with the whole referral system in the district and not just a delay in the hospitals because it’s possible that completion of some of the partographs had begun in health centres before referral of clients to hospitals. This view is supported by the results of the promptness of emergency caesarean sections which showed that for 76% of emergency caesarean sections, the baby was delivered within one hour after deciding to perform the procedure. There is some evidence that maternal and newborn outcomes are more likely to be positive if the interval between decision for emergency caesarean section and delivery of the baby is less than 30 minutes [44].

A shortage of midwives in the district was noted. Only half of the minimum required number of midwives was present. When the number of skilled attendants at birth is inadequate, the quality of services is likely to be poor and utilisation will consequently fall. Inadequate staffing, low remuneration and high workload heavily affect quality of care [27,45]. Various strategies are currently being implemented to attract and retain staff in the region [11]. Assessment of the maternal and neonatal care knowledge of maternity staff revealed knowledge gaps for the effective delivery of safe motherhood services. Similar findings have been documented in a study conducted in Soroti; a nearby district [46]. Quality of care can also be linked to availability of physical infrastructure. Physical facilities for maternity care were missing in some facilities with health centres being the most disadvantaged. Lack of infrastructure at health facilities can not only compromise the actual quality of care delivered but also the perceived quality among users, resulting in the underutilisation of services.

This study has a number of limitations. Firstly, the absence of the observation of routine procedures of MNH care delivery is an issue. There may be differences between what people/health workers say they did, and what they actually did. Similarly, when investigating which services women had received, we asked mothers whether they had received services (or checked their files/records). In both cases, this may not be a true reflection of what really happened. Nevertheless, studies done elsewhere have shown that women are good at recalling pregnancy related events [47,48] and hence the effect of these potential limitations on our findings is thought to be minimal. Secondly, women who delivered at some of the HCs were discharged with their partographs and files. Assessment of the quality of normal delivery care at these facilities was therefore limited to the information available in delivery registers. In addition, the average duration of service of staff was obtained by dividing the number of person months at each duty station by the number of staff whose data on duration of service at the duty station was available. This might have resulted in an undercount. Whereas this study was conducted in only two districts, other districts in Karamoja region could benefit from the study’s recommendations. There is however a need for further assessments on the availability, utilisation and quality of MNH care services in Karamoja at different levels of health units. This would improve our understanding of the observations presented in this paper, and consequently advance policy-relevant information to decision-makers at all levels.

## Conclusions

This study shows that there were serious challenges to the delivery of maternal health services in Karamoja region. There is an urgent need to upgrade some HC IIIs to meet BEmOC standards. Second, the low coverage of ANC demands an increase in the current efforts to deliver this service. Efforts currently in place (for example food distribution linked to antenatal visits) should be sustained. Thirdly, because the quality, effectiveness, efficiency, accessibility and viability of health services depend mainly on the performance of those who deliver them, staff should be re-oriented on various aspects of maternal and neonatal care and on the importance of providing the required standard of care [49]. This requires the allocation of more resources to recruit skilled personnel and to facilitate a performance improvement process to address the burden of maternal mortality in Karamoja. Monitoring the EmOC relies on proper recording and record keeping. These aspects need to be strengthened at all the health facilities. Lack of equipment, drugs, supplies and poor infrastructure seriously compromise the quality of care provided. With most deliveries taking place at home, there is need to focus efforts on the demand side. Involving TBAs by encouraging them to refer women to health facilities for delivery has the potential to produce good results [24]. Finally, missed opportunities for delivering health messages to mothers attending ANC were noted. Maternity staff can benefit from having a checklist of key messages they need to share with antenatal and postnatal mothers [50]. Health promotion should also focus on dealing with cultural barriers of delivery in health units and increasing knowledge of the danger signs of pregnancy amongst women. In conclusion, in order to be successful in reducing maternal and newborn morbidity and mortality in this and other areas in Uganda with similar socio-economic profile, there is need to increase availability and accessibility of skilled attendants at birth, address the low utilisation of maternity and postnatal services, and improve the availability and accessibility of EmOC services, with particular attention to BEmOC services.

## Abbreviations

AIDS: Acquired immunodeficiency syndrome
ANC: Antenatal care
APGAR: Appearance, pulse, grimace, activity and respiration
BEmOC: Basic emergency obstetric care
CEmOC: Comprehensive emergency obstetric care
CUAMM: *Collegio universitario aspiranti medici missionari*
EmOC: Emergency obstetric care
HC: Health centre
HIV: Human immunodeficiency virus
IPT: Intermittent preventive therapy
MDG: Millennium development goal
MMR: Maternal mortality ratio
MNH: Maternal and neonatal health
PMTCT: Prevention of mother to child transmission
PNC: Postnatal care
PPH: Postpartum haemorrhage
TBA: Traditional birth attendant
